# Use of Adrenalin during Ether Anesthesia

**Published:** 1907-09

**Authors:** 


### ABSTRACTS.

### Use of Adrenalin During Ether Anesthesia.

CHARLES S. VENABLE, Charlottesville, Va., says: recognising that my experience (*Virginia Medical Semimonthly,* February 22, 1907), in the use of adrenalin during ether anesthesia is but very limited, covering a course of only eighteen cases, and knowing the many fallacies attendant upon too early conclusions, I feel a great hesitancy in making this report. However, owing to the uniform result that has attended its use, I am prompted to do so now.

I found that a 25 per cent. aqueous solution of the standard 1 in 1000 gave the best results, and that by first pouring ether in the towel cone and spraying the adrenalin solution on it, depending on the ether to vaporise it sufficiently for inhalation, was the best mode of administration. Three to six minute intervals are sufficient for its use and a total of from one-half to one ounce of this solution is enough for an operation lasting from thirty minutes to an hour. The effects are a more uniform etherisation, the pulse becoming steadier, slower and of better character more rapidly than under ether alone; respirations are quiet and regular, the bronchial secretions are practically checked, and the progress of the operation is not interrupted.

These cases were not selected, and among them were old alcoholics; two women over sixty, one of them nearly eighty years of age. Three were very long tedious operations, lasting over two hours, and in none of the series was any stimulation required during the anesthesia. Recovery from the anesthetic was uniformly good; there was practically no post-operative shock, and no stimulation was needed in any one of the cases; only two patients vomited at all and very little nausea was complained of.

From the foregoing facts I conclude that owing to the contraction of the smaller vessels the bronchial glands secrete less mucus, and there is better aeration in the bronchioles and pulmonary vesicles, less ether is required to produce anesthesia and there is less probability of ether pneumonia following. The adrenalin, acting generally from absorption, is a powerful stimulant; it materially lessens shock, lessens the capillary ooze at the field of operation, and is of great benefit to the much weakened patient.

